# Supplementary material for: Plasma Levels of Propionylcarnitine Improved Prediction of Heart Failure and All-Cause Mortality in Patients with Stable Coronary Artery Disease
Source: Biomolecules. 2024 Dec 29;15(1):27. doi: 10.3390/biom15010027 (PMC11764408; doi:10.3390/biom15010027)
Supplement: Supplementary file 1 [file biomolecules-15-00027-s001.zip › biomolecules-3330540-supplementary.pdf]

Supplementary Materials:

Table S1. Goodness-of-fit and discrimination capability comparison of the models. (a) The values of -2Log Likelihood, Nagelkerke's R^2, and the Likelihood ratio test ($LR\chi^2$) p-value. Also, the DeLong's test, and the IDI (integrated discrimination improvement), NRI (net reclassification index) p-values. *Comparisons against Model C. **(b)** The sensitivity and specificity for cut-off probabilities for the ROC analyses.

Model		HF or death					
		**-2 log Likelihood**	**Nagelkerke R2**	**LRχ 2 (p-Value)**	**DeLong's test (p-Value)***	**IDI (p-Value)***	**NRI (p-Value)***
A	NGAL (x10^2 ng/mL) / C3:0 (x10^{-2} μM	107.72	0.25	<0.001	0.011	0.001	0.022
B	Gal-3 (ng/mL)<br>NGAL (x10^2 ng/mL) / C3:0 (x10^{-2} μM)	94.27	0.38	<0.001	0.2	0.101	0.160
C	Age (years)<br>Gal-3 (ng/mL)<br>NGAL (x10^2 ng/mL) / C3:0 (x10^{-2} μM)	83.576	0.47	<0.001	-	-	-

(a)

Model		ROC analyses			
		**Cut-off**	**Sensitivity**	**Specificity**	**p-Value**
A	NGAL (x10^2 ng/mL) / C3:0 (x10^{-2} μM	0.22	0.64	0.83	<0.001
B	Gal-3 (ng/mL)<br>NGAL (x10^2 ng/mL) / C3:0 (x10^{-2} μM)	0.24	0.76	0.9	<0.001
C	Age (years)<br>Gal-3 (ng/mL)<br>NGAL (x10^2 ng/mL) / C3:0 (x10^{-2} μM)	0.17	0.8	0.81	<0.001

(b)

Table S2. Multivariable Cox regression for relative risk of HF or all-cause death. Model "A", represents the initial model obtained by using the forward stepwise method (NGAL/C3:0), and model "B", reflects the incorporation of Gal-3 into the previous model. The hazard ratio (HR) with 95% confidence interval (CI), p-value, and C-statistic are exposed for each model. C3:0, propionylcarnitine; Gal-3, galectin-3; NGAL, neutrophil gelatinase-associated lipocalin.

Models		HF or death			
		**HR**	**(95% CI)**	**p-Value**	**C-Stat**
A	NGAL (x10^2 ng/mL) / C3:0 (x10^{-2} μM)	4.52	2.749-7.428	**<0.001**	0.75 (0.620-0.872)
B	Gal-3 (ng/mL)	1.28	1.21-1.458	**<0.001**	0.85 (0.772-0.933
	NGAL (x10^2 ng/mL) / C3:0 (x10^{-2} μM)	3.48	2.031-5.974	**<0.001**	
